# A systematic review and thematic synthesis on the experiences of accessing and attending psychological therapy for informal carers of people living with dementia

**DOI:** 10.1186/s12877-025-05986-7

**Published:** 2025-05-19

**Authors:** Jacquelyn Yang, Caroline Fearn, Amber John, Sarah Hoare, Seryn Chang, David Zammitt, Joshua Stott

**Affiliations:** 1 ADAPT Lab, UCL Department of Clinical, Educational and Health Psychology, Division of Psychology and Language Sciences, University College London, 1-19 Torrington Place, London, WC1E 7HB UK; 2 Psychology Department, University of East London, Docklands Campus, University Way, London, E16 2RD UK; 3 Department of Psychology, University of Liverpool, 1 Old Hall Street, Liverpool, L3 9GH UK; 4 UCL Research Department of Clinical, Educational and Health Psychology, Division of Psychology and Language Sciences, University College London, 1 – 19 Torrington Place, WC1E 7HB London, UK

**Keywords:** Dementia caregivers, Psychological therapy, Systematic review, Thematic synthesis

## Abstract

**Background:**

Informal carers of people living with dementia are at a higher risk of experiencing mental health difficulties than the general population, yet many are not able to access timely psychological support and their psychological needs are often overlooked. It is therefore important to develop a greater understanding of carers’ lived experiences in accessing and attending psychological therapy, to help tailor therapies to meet their individual needs. To our knowledge, this is the first thematic synthesis of qualitative literature on carers’ experiences of accessing and taking part in psychological therapies.

**Methods:**

Three databases were systematically searched for qualitative literature, and 23 studies were included. Their quality was assessed and the data extracted was included in the thematic synthesis.

**Results:**

Findings were organised into five overarching themes: i) Mental health and relationship difficulties (context); ii) Overall positive experiences of therapy (including specific techniques, therapist factors and therapeutic relationship, social support); iii) Common changes experienced (e.g. increased awareness of one’s emotions and needs, increased self-care and self-compassion); iv) Unhelpful experiences of therapy, suggestions and further needs; and v) Impact of wider societal contexts and events.

**Conclusions:**

Given the predominantly positive experiences of therapy and mechanisms of change described, findings suggest that psychological therapies can be helpful for carers of people living with dementia. Additionally outlined are specific techniques to tailor therapy (regardless of approach) to best meet carers’ needs, and suggestions for improvement. Future research should try to understand for whom and under what circumstances (e.g. wider contexts) psychosocial interventions become most effective in this population.

**Supplementary Information:**

The online version contains supplementary material available at 10.1186/s12877-025-05986-7.

## Introduction

Dementia is a global public health priority and in 2015, the global annual cost of dementia was estimated to be US$818 billion [1]. Informal unpaid carers (henceforth ‘carers’), usually family members, play a significant role in dementia care and support for people living with dementia (PLWD), and represent approximately 40% of the total cost of dementia globally [2, 3]. The annual global number of informal care hours provided to PLWD living at home was equivalent of more than 40 million full-time workers in 2015, which is estimated to increase to 65 million by 2030 [3].

Caring for PLWD can involve emotional, physical, and financial strains. Carers often experience high levels of stress, anxiety, and depression due to the demands of caregiving as well as witnessing the decline in their loved one's cognitive abilities [4, 5]. This strain can lead to negative consequences for both carers and care recipients, including: reduced quality of life; increased healthcare service use and institutionalisation [6, 7] and 55% higher mortality risk for those who reported higher caregiver strain [5].

Psychosocial interventions can improve and maintain carer wellbeing [8, 9]. Mindfulness Based Stress Reduction (MBSR), Mindfulness-based Cognitive Therapy (MBCT), Cognitive Behavioural Therapy (CBT), and Acceptance and Commitment Therapy (ACT) have been shown to reduce caregiver depression and burden [4, 10, 11]. Psychoeducation, skill-building, psychotherapy, counselling, and multicomponent interventions can also improve carer emotional well-being [10, 12].

Despite the efficacy of such interventions, many carers are not able to access timely psychological support due to various barriers, such as difficulties in prioritising their own health needs, reduced mobility, geographical location, lack of respite care, and long waiting lists due to a shortage of therapists [13–16]. Carers’ psychological needs are frequently overlooked within healthcare systems, particularly concerning access to, and attendance in, psychological therapy. Reviews highlight several needs: to address carers’ physical and psychological health; to manage carers’ own personal lives [17, 18], and for a person-centred approach to care planning that accounts for the needs of the carers, to promote better caregiver well-being and quality of life [19]. Carers’: access to emotional support; information and respite care; and adjustment to caregiver identity, should be reviewed as part of the care package for the PLWD.

### Aims and rationale

Given the mental health support needs of carers of PLWD, it is important to develop a greater understanding of carers’ lived experiences in accessing and attending psychological therapy, in order to address the challenges of treatment accessibility and tailoring for this group [13]. Psychological (talking) therapy interventions (henceforth ‘psychological therapies’) is defined as “treatments for mental and emotional problems like stress, anxiety and depression”; which involve talking to a trained and accredited professional [20]. Understanding such experiences is crucial for improving mental health services as well as outcomes for both carers and their care recipients. While quantitative studies have provided valuable insights into the effectiveness of psychological interventions for carers of PLWD [8, 10, 12, 21], qualitative research offers a deeper understanding of their lived experiences, perceptions, and the contextual factors influencing their access to and engagement with therapy. The Medical Research Council (MRC) framework [22] highlights the importance and benefit of process evaluation of psychological interventions and understanding how interventions contribute to change, including how they interact with their context and wider dynamic systems.

To our knowledge, there are no existing reviews which specifically address the views and experiences of carers of PLWD in accessing and attending psychological therapy. This review seeks to address this gap and inform the development of tailored person-centred interventions and support services to better meet their psychological support needs. Review aims are to:

- Identify existing qualitative literature on carers of PLWD’s views and experiences in accessing and/or taking part in psychological therapies.
- Synthesise findings and outline the associated implications, including recommendations for policymakers, healthcare professionals and service providers, to improve access to and experience of psychological therapy interventions for carers of PLWD.

## Methods

We conducted a systematic review and thematic synthesis to address our aims. A protocol was registered on PROSPERO (CRD42023397470). Findings are reported in accordance with the Preferred Reporting Items for Systematic Reviews and Meta-Analyses (PRISMA) guidelines [23].

### Eligibility criteria

This review focused on studies exploring the experiences of carers of PLWD (any type of dementia) regarding the access and provision of psychological therapies (e.g., Cognitive Behavioural Therapy, Acceptance and Commitment Therapy, Counselling, Mindfulness). The inclusion criteria were:

- Peer-reviewed empirical studies
- Studies that used a qualitative analytical approach (i.e. had to specify a formal method of qualitative analysis or include a reference to a qualitative analysis process) including where a qualitative element was conducted within a mixed methodology approach.
- Studies investigating carers experiences of, or access to psychological therapy were included. This also included the following:
  - If the therapy involves both the PLWD and carer, studies where therapy focuses on only carer wellbeing/mental health rather than the PLWD were included (i.e. excluding those where therapy focuses solely on the PLWD’s wellbeing).
  - Web- or app-delivered interventions which are guided by a human or include a human component, as well as in-person or telephone-delivered interventions.
  - Studies that focused on the delivery of psychological therapies with adult carers of PLWD, including investigating the lived experiences of these carers in accessing and taking part in such therapies.
  - Studies that contained themes related to psychological therapies within a wider piece of analysis, including feasibility studies with qualitative components (where qualitative analysis was conducted as mentioned above).
  - Studies that contained themes related to psychological therapies which had been delivered within the context of broader intervention packages (e.g. multi-component psychosocial interventions).
  - Studies that explored or included the perspectives of relevant others who were not carers (e.g. healthcare professionals), only if these individuals discussed their views on carers’ experiences of psychological therapy. This was deemed of particular relevance given that these individuals may also have been involved in the provision and delivery of therapy, and may have received feedback from carers about their experiences that could help inform service provision through multiple perspectives.
  - Carer needs-based studies that include participants (carers) who have accessed, are attempting to access or are considering accessing psychological therapy.

Exclusion criteria were:

- Case studies.
- Studies on adolescents or children (under 18).
- Non-English language (due to a lack of available translation resources).
- Systematic reviews or narrative reviews.
- Studies of-guided web-based self-help resources.
- Studies where the intervention only involves psychoeducation or support groups.
- Studies which focus exclusively on physical healthcare or on access and provision of supports which do not involve any element of psychological therapy.
- Studies that involve co-production of a psychological therapy with no mention of participants’ prior/current experience of therapy.

### Search strategy and selection criteria

Three electronic databases were searched in October 2023 (Web of Science database; and PsycINFO and CINAHL databases via Ovid). The search strategy (Appendix A) was adapted from that of a related systematic review on the experiences of PLWD in accessing and taking part in psychological therapies (Zammitt D: The experience of individuals with dementia in accessing and taking part in talking therapies for mental health difficulties: a systematic review and thematic meta-synthesis, unpublished doctoral dissertation). Search terms for psychological therapy were based on: therapies offered by the National Health Service in the UK [20], specifically those listed in the NHS Talking Therapies for anxiety and depression Manual [24]; those shown in existing literature; as well as generic talking therapy and psychotherapy terms. Studies were not restricted based on date of publication. Search terms included text keywords, subject headings and MeSh terms where relevant.

**Fig. 1:**
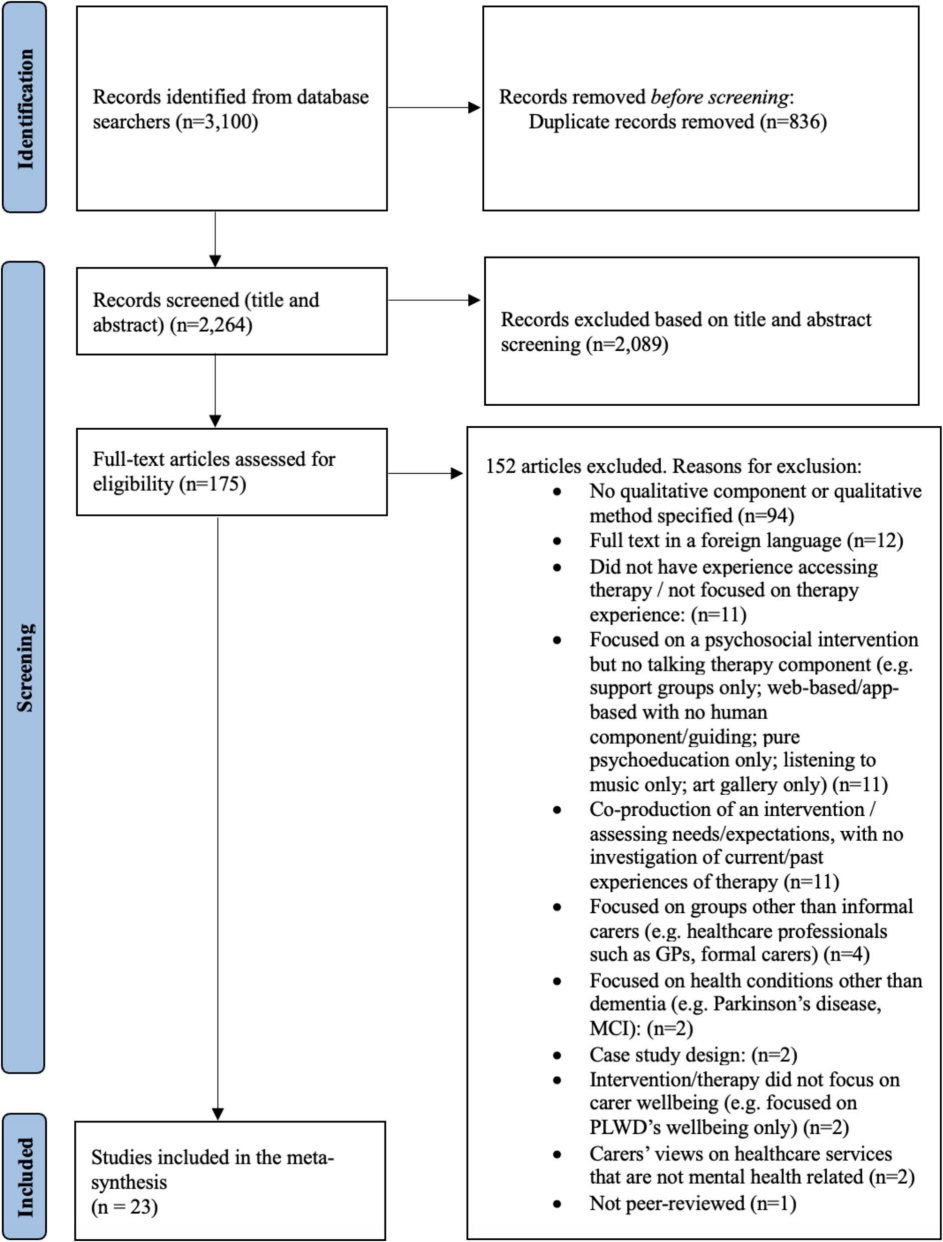
PRISMA Flow Diagram [23]

Search results were combined and imported to Rayyan [25]. First author JY manually identified and removed duplicates. JY then screened the titles and abstracts of the remaining studies, using the predefined inclusion and exclusion criteria. 10% of the title and abstracts were blindly screened by SC, with 99% agreement. Discrepancies were addressed through discussion. Subsequently, the full texts of the remaining studies were screened by JY, and reasons for exclusion were recorded for each excluded study (Fig. 1). 10% of the full texts were also screened by JS, and any discrepancies were discussed until consensus was reached.

### Quality assessment

Long et al.’s [26] revised version of the Cochrane recommended [27] Critical Appraisal Skills Programme (CASP) Checklist for qualitative research (Critical Appraisal Skills Programme, 2018) was used to carry out quality assessment of included studies. (items used are available in Appendix B.)

For mixed-methods studies, only the qualitative components were rated. Furthermore, following Long et al.’s [26] guidance, deciding criteria were used to establish relative study quality for the current review’s included studies. Three “tipping point” criteria were considered priority: 1) That the qualitative methodology is appropriate and relevant for addressing the research goal (e.g. if research seeks to illuminate/interpret the subjective experiences of participants); 2) That the research design is appropriate to address the aims of the research; and 3) That the studies had performed robust and comprehensive data analysis, to maximise the contribution our review could make to current understanding, and consider trustworthiness or credibility of the findings.

The CASP Checklist does not define a scoring system [28].Therefore, a score was obtained for each study based on the proportion of ‘tipping point’ items [26]; and high, medium and low-quality classifications were determined relative to all the included papers in the present review. Studies were classified as “high” if the study had all three ‘yes’ “tipping point” items; “medium” if the study had 2 ‘yes’ “tipping point” items; and “low” if the study had one or less ‘yes’ “tipping point” item. JY appraised all 23 studies. SC also independently appraised > 10% (three) of the studies. Any discrepancies were discussed until consensus was reached.

### Synthesis of results

Thematic synthesis was conducted using Thomas and Harden’s [29] framework which was specifically designed for synthesising qualitative research. Following Thomas and Harden’s [29] guidelines and considering the lack of existing qualitative research in this area, the data was analysed inductively.

Digital versions of the studies were used as primary data sources. As recommended by Thomas and Harden [29], all text categorised as ‘results’, ‘findings’, or ‘discussion’ from the papers was extracted and uploaded to NVivo [30]. The current review used Long et al. [26]’s adaptation of Thomas and Harden's [29] approach, whereby higher quality studies were prioritised and coded first and lower quality ones were coded afterwards. Therefore codes from higher quality studies formed the foundation of the thematic framework and carried more weight in this study's findings.

First, JY read and re-read the 23 papers, to familiarise and immerse herself with the data and to start forming initial codes. Then coding was carried out on data relevant to the research question from the five papers determined as “high” quality. First-order data were coded first where possible, followed by second-order data.

SC independently followed the same process for two of the “high” quality papers, shared the initial codes with JY who then integrated resulting codes into a combined coding framework. JY subsequently re-read the studies of “medium” quality; revising or adding codes from the initial framework, where appropriate. This process was repeated for the studies which were appraised as “low” quality, and as a result of this no new codes developed. Initial descriptive themes based on code patterns were developed and then formed into analytical higher-order themes.

## Results

### Study selection

Searches across the three databases identified a total of 3,100 records. 2,264 remained after de-duplication, 175 studies were retrieved for full-text screening and 23 studies were included in the thematic synthesis. See Fig. 1 for PRISMA flow diagram.

### Study characteristics

Overall, there were 14 qualitative studies and nine mixed-method studies. Most studies (*N* = 16) were published between 2014 and 2023 (nine between 2020 and 2023). 10 were conducted in the United States, four in the UK, two in Germany, and one each from Denmark, Canada, Australia, Norway, The Netherlands, Hong Kong China, and Sweden. Most (*N* = 17) focused solely on carers; three studies included both carers and PLWD; one included both carers and professional; and two focused solely on the views of professionals (and two focused solely on the views of professionals (i.e. novice therapists or counsellors).

There were two cases where two studies reported on the same intervention [13, 31–33]. In one of these cases [32, 33] but not the other [13, 31], participants may have overlapped, although how is not specified in the papers, and thus both were included. Thus overall, 700 carers 27 professionals (including therapists, care facility staff, and counsellors), and five carer and PLWD dyads are represented in the results.

Five studies explored counselling interventions, four explored mindfulness-based approaches, four examined CBT/Cognitive behavioural interventions (CBI), four explored multi-component interventions, two examined the same ACT-based intervention, one explored Mentalizing Imagery Therapy and one involved telephone support with specific techniques.

Seven studies used content analysis; six used thematic analysis; six used a grounded theory approach and two used framework analysis. One described using an “inductive, iterative” approach; and one described using a “within-case analysis” and “cross-case theme analysis”.

See Table 1 for details of all characteristics.

**Table 1:** Summary of included studies

Study ID	First author, Year, Location	Main focus	Method	Participant group(s)	Participant gender	Participant age (mean)	Participant ethnicity	Participant mental health information	PLWD’s dementia diagnosis	Intervention approach & modality	Quality Rating descriptor
1	Contreras (2022), UK [13]	Carers’ views and acceptability of internet-delivered, therapist-guided, self-help ACT for family carers of PLWD (iACT4 CARERS)	Qualitative; Semi-structured interviews. Thematic Analysis	23 unpaid primary carers: Spouses (*n* = 12), Adult children (*n* = 10), Sibling (*n* = 1)	Female (*n* = 20), Male (*n* = 3)	62	Not reported	Mild- moderate depression or anxiety, as indicated by GAD-7 or PHQ-9 scores	AD (*n* = 6), VD (*n* = 3), DwLB (*n* = 1), FTD (*n* = 1); Mixed dementia (*n* = 9), Other (*n* = 3)	Internet-delivered, therapist-guided, self-help ACT for carers of PLWD (iACT4 CARERS). 8 individual online sessions, and optional 3 online peer-support groups	High
2	Contreras (2021), UK [31]	Therapists’ views and acceptability of providing internet-delivered, therapist-guided, self-help ACT for family carers of PLWD (iACT4 CARERS)	Qualitative; Semi-structured interviews. Thematic Analysis	8 novice therapists	Female (75%), Male (25%) (percentages reported only)	Not reported	Not reported	Not reported	Not reported	Internet-delivered, therapist-guided, self-help ACT (iACT4 CARERS) 8 individual online sessions, and optional 3 online peer-support groups	High
3	Sørensen (2008), Denmark [34]	Participants’ experienced outcome of an intensive structured psychosocial intervention programme for home-living patients with mild AD and their spousal carers	Qualitative; Semi-structured interviews Grounded theory approach (Dellve, Abramsson, Trulsson, & Hallberg, 2002; (Crabtree & Miller, 1999)	Partner carers (*n* = 10), PLWD (*n* = 10)	PLWD: Female (*n* = 5), Male (*n* = 5) Carers: Female (*n* = 5), Male (*n* = 5)	Carers: 73.4, PLWD: 66.7	Not reported	Not reported	PLWD described as having ‘Mild AD’	Multicomponent 6-month intervention, which included 5 main components: 1) Tailored counselling for the individuals and the family, 2) Education course for groups of PLWD, 3) Education course for carers, 4) Outreach telephone counselling, and 5) Log book kept by PLWD and carers separately	High
4	Tahsin (2021), Canada [35]	Carers’ experience of a single mindfulness session and the feasibility of mindfulness to be integrated into their daily lives	Qualitative; Semi-structured interviews. Thematic analysis	6 carers (relationship to PLWD not reported)	Female (*n* = 5), Male (*n* = 1)	Range: 60–69 (mean not reported)	Not reported	Not reported	Not reported	Single 15-min group mindfulness session	High
5	Elvish (2014), UK [36]	Carers’ experiences of receiving counselling/psychotherapy	Qualitative; Semi-structured interviews. “Narrative methodology (Williams & Keady, 2008) and the thematic approach of ‘holistic-content’ analysis (Lieblich, Tuval-Mashiach, & Zilber, 1998)	6 carers: Spouses (*n* = 4), Adult children (*n* = 2) At the time of interview, five participants were actively undertaking therapy, and one participant had recently completed their sessions	Female (*n* = 5), Male (*n* = 1)	Range: 55–80 (mean not reported)	All White/British	Not reported	Not reported	Individual counselling/psychotherapy, eclectic approach including humanistic, integrative, person-centred, non-directive, personal construct, acceptance and commitment, cognitive- behavioural, schema, and psycho-educational approaches	High
6	Brooks (2022), Australia [37]	Explored long-term care placement transitional support needs and preferences of spousal dementia carers (from spousal carer and long-term care facility staff perspectives), to inform subsequent support and intervention development	Qualitative; Semi-structured interviews or small group discussions held separately with carers and care facility staff. Framework approach (Ritchie & Spencer, 1994)	Spousal carers (*n* = 9), Care facility staff (*n* = 11) Psychological support had been sought by some spouses experiencing high levels of grief or depression, from counsellors or psychologists	Carers: Female (*n* = 8), Male (*n* = 1)	Range 60 s-80 s (mean not reported)	Caucasian, Australian or British by birth. No other information reported	Not reported	Not reported	Not specified, as reported on the transitional support needs of participants	Medium
7	Griffiths (2020), UK [38]	Perspectives of PLWD and their family carers on a relational counselling intervention delivered through a third sector organisation within England	Qualitative; Semi-structured interviews. Framework analysis	23 Carers: Spouses (*n* = 11), Adult children (*n* = 12) PLWD (*n* = 6) This included three ‘dyads’, whereby both the person with dementia and their respective caregiver attended sessions	Carers: Female (*n *= 19), Male (*n* = 4) PLWD: Female (*n* = 3), Male (*n* = 3)	Carers: Not reported, PLWD: 81	Carers: White British (*n* = 22); Black British (*n* = 1) PLWD: White British (*n* = 4); White European (*n* = 2)	Not reported	AD (*n* = 4), VD (*n* = 1), Mixed dementia (*n *= 1)	12-week relational counselling intervention. Most participants attended sessions individually, with three attending as ‘dyads’ (PLWD and their carer attended together)	Medium
8	Johannessen (2015), Norway [39]	Family carers'experiences of attending a multicomponent psychosocial intervention program for carers and PLWD, and participants’ advice on how to develop the intervention program	Qualitative; Semi-structured interviews. Content analysis (Corbin & Strauss, 2008)	20 family carers: Spouses (*n* = 13), Adult children (*n* = 7)	Female (*n* = 15), Male (*n* = 5)	64.5	Not reported	Not reported	Not reported	Multicomponent psychosocial intervention which includes individual counselling, group meetings, cognitive techniques such as structured problem solving, and education	Medium
9	Kazmer (2018), United States [40]	Experiences of dementia family carers who participated in an integrative, cognitive-behavioural and spiritual counselling intervention, and also experiences of FCNs who delivered the intervention. Including their views on the benefits and drawbacks of the intervention	Qualitative component reported only; Semi-structured interviews “An inductive, iterative process of generating coding that was collaborative with the research team” (Ahuvia, 2001;Bradley, Curry, & Devers, 2007; Meyer & Avery, 2009; Steinke, 2004)	7 carers (relationship to PLWD not specified)	Female (*n* = 6), Male (*n* = 1)	Not reported	Not reported	Not reported	Scored 10 or more on the PHQ-9	Cognitive-behavioural and spiritual counselling (CBSC) intervention, delivered by FCNs who had a subspecialty in faith-based service provision. The intervention included 12 1-h individual sessions delivered biweekly in carers’ homes. Counselling focused on identified problems of the carers, and the most frequently used CBT elements were relaxation training, cognitive reframing, increasing assertiveness, and building pleasant daily Spiritual counselling focused on the use of religious coping strategies, such as prayer, meditation, positive affirmation, and communal support	Medium
10	Lee (2022), United States [41]	Experiences of family carers of PLWD in receiving a telephone support intervention for diverse family carers of PLWD during the COVID-19 pandemic	Qualitative; Data were collected through detailed call logs by the Research Assistants Thematic Analysis	23 family carers: Spouses (*n* = 11), Adult children (*n* = 9)	Female (*n* = 20), Male (*n* = 3)	60.2	Hispanic (*n* = 8), Non-Hispanic White (*n* = 7), Korean (*n* = 5), Vietnamese (*n* = 3)	AD (*n* = 17), VD (*n* = 2), DwLB (*n* = 1), Unsure (*n *= 3)	Not reported	Four-week individual telephone support intervention which included psychoeducation, stress management and compassionate listening, described as being “culturally and linguistically appropriate”	Medium
11	Berk (2019), Netherlands [42]	PLWD and their partners’ views of a mindfulness- based intervention	Mixed methods: Semi-structured interviews. Deductive content analysis	Partner carers (*n *= 7), PLWD (*n *= 7)	Carers: Female (*n *= 5), Male (*n *= 2) PLWD: Female (*n *= 2), Male (*n *= 5)	Carers: 70.75 PLWD: 71.46	Not reported	AD (*n *= 4), VD (*n *= 2), FTD (*n *= 1)	Not reported	Attention Training for PLWD and their carers (TANDEM), which consisted of eight weekly group sessions of 2.5 h each, a 4 h silent day and daily homework assignments of 45 min per day. A range mindfulness of exercises were taught and psycho education about stress and communication	Medium
12	Yang (2023), United State [43]	Experiences and perceived benefits of family dementia carers who underwent MIT	Qualitative; Semi-structured interviews. A “collaborative approach (Richards & Hemphill, 2018)”, including a within case analysis and a cross case theme analysis (Ayres et al., 2003)	11 family carers: Spouses (*n *= 4), Adult children (*n *= 7)	Female (*n *= 9), Male (*n *= 2)	Not reported. “Six were over the age of 65, while the other five were between 38 and 65”	Caucasian (*n *= 7), African American (*n *= 2), Hispanic (*n *= 2)	Not specified	Scored 10 or more on the PHQ-9	4-week MIT program, which included group sessions of mindful stretching, breathing exercises, group discussion, and guided imagery	Medium
13	Glueckauf (2012), United States [33]	Explored carers’ experiences of a telephone-based CBT for African American dementia carers with depression	Mixed-methods; Semi- structured interview. Grounded theory approach, open-coding procedure (Charmaz, 2006; Strauss & Corbin, 1998)	11 carers (relationship to PLWD not specified)	Female (*n *= 10), Male (*n *= 1)	58.09	All African American	AD (*n *= 5), DwLB (*n *= 2), VD (*n *= 2), Dementia of unknown etiology (*n *= 2)	Mean PHQ-9 score within the moderate range of depression (M = 13.0, SD = 2.72)	Telephone-Based CBT. The intervention program consisted of a total of 12, 1-h, weekly sessions, 7 group and 5 individual carer goal-setting and implementation sessions	Medium
14	Vernooij-Dassen (2010), United States [44]	Explore the provision of the New York University Caregiver Intervention (NYUCI), family-centred counselling, to enhance its implementation	Qualitative; Group interviews. Grounded theory approach	8 counsellors who provided the intervention	All female	Ranged from “mid- 60 s to 95 years” (mean not reported)	Not reported	Alzheimer’s only	Not reported	The New York University Caregiver Intervention (NYUCI) expands the intervention to all participating family members and focuses on the family context and includes individual and family counselling and support groups	Medium
15	Gaugler (2018), United States [45]	A process evaluation of the New York University Caregiver Intervention-Adult Child (NYUCI-AC) to describe its delivery and determine which of its components were associated with key outcomes (caregiver stress and well-being; care recipient residential care admission)	Mixed methods; Survey, qualitative component included one open-ended feedback question. Thematic Analysis	54 Adult children carers participated in the overall study, and only 25 participated in the qualitative component	Female: 88.7%. (percentage reported only)	51.23	Caucasian: 94.4% (percentage reported only)	Alzheimer’s only	Caregiver Perceived Stress Scale: M = 15.07, Geriatric Depression Scale: M = 6.11, Quality of Life: Cantril Ladder: M = 74.63	Multi-component psychosocial intervention (the NYUCI) and includes individual and family counselling and support groups	Low
16	Gräßel (2010), Germany [46]	Predictors for utilisation and expected quality of counselling from a family carer’s perspective. Specifically asked which variables of the care situation, the caregivers and their attitudes act as predictors for the utilization of caregiver counselling; and what are the views of caregivers about the quality of caregiver counselling	Mixed-methods; survey, qualitative component included one open question Content analysis (Morgan, 1993)	404 carers: Spousal (43.8%), Adult children: (43.9%), Others (7.3%) (percentages reported only) 155 of the family carers (38.4%) were “users of counselling”	Female: 73.3% (percentage reported only)	61.3	Not reported	Not specified but appears to be AD only	Not reported	Not specified, as reported on counselling needs of participants	Low
17	Glueckauf (2022), United States [47]	An initial evaluation of the quantitative and qualitative outcomes of the African American Alzheimer’s Caregiver Training and Support Project 2 (ACTS2). Qualitative objectives included examining carers’ perceptions of the effectiveness of in-session training activities, quality of relationships among group participants and their facilitator, and appraisals of spiritual elements of the program	Mixed-methods; Qualitative component was semi-structured interviews Inductive and Grounded theory approach with open coding (Charmaz, 2006)	9 carers: Adult children (*n *= 6), Spouse (*n *= 1), Adult grandchild (*n *= 1), Niece (*n* = 1)	Female (*n *= 8), Male (*n *= 1)	56.0	All African American	All AD	Scored 10 or above on the PHQ-9	ACTS2 was lay pastoral care facilitator-led (trained lay faith community worker-led), faith-integrated telephone CBI for African American dementia carers with moderate depression. The 12-week training included 7 skills-building groups and 5 individual problem solving sessions; and integration of spiritual elements such as prayer	Low
18	Hoppes (2012), United States [48]	The effects of a brief course of mindfulness training on the well-being of individuals caring for family members with dementia	Mixed-methods; qualitative component consisted of interviews. Thematic analysis using grounded theory’s open coding stage (Charmaz, 2006; Glaser & Strauss, 1968; Strauss & Corbin, 1998)	11 family carers: Spouses (*n *= 7), Adult children (*n *= 4)	Female (*n *= 10), Male (*n *= 1)	63.8	All Caucasian	Not reported	Not reported	4 group sessions (1-h per session, once a week for 4 consecutive weeks) of mindfulness training, based on MBSR	Low
19	Kazmer (2013), United States [32]	Used qualitative interview data from the African-American Alzheimer's Caregiver Training and Support (ACTS) project (Glueckauf et al., 2012) to understand information problems, settings, and uses of dementia carers, over the course of CBT for depression	Secondary qualitative data analysis; Interview data from semi-structured interviews. Open coding approach (Strauss & Corbin, 1998)	16 carers	Not reported	Not reported	All African American	Not reported	All AD	The intervention focused on CBT skills and enhancing social support. 12, 1-h, weekly sessions, 7 group and 5 individual goal-setting and implementation sessions	Low
20	Kor (2019), Hong Kong China [49]	Feasibility and preliminary effects of a modified MBCT for family carers of PLWD	Mixed-methods; qualitative component involved a focus group (semi-structured interview). Content analysis	8 carers participated in the focus groups: Adult children (*n *= 5), Spouses (*n *= 3)	In overall study: Female (*n *= 30), Male (*n *= 6) (breakdown not reported for focus group participants only)	56.9	Not reported	Not reported	Not reported	A group-based, 10-week, 7-session modified MBCT program included mindfulness activities (e.g. mindful walking, body scanning, mindful eating), psychoeducation on caregiving, and group sharing	Low
21	Koufacos (2023), United States [50]	Results of a VHA project, whereby carers received individual assessment and counselling focused on stress-reduction	Mixed-methods; survey, qualitative component was open-ended questions Content analysis	24 out of 30 carer participants completed the survey (relationship to PLWD not reported)	Females: 92% (percentage reported only)	Over the age of 65: 75% (mean not reported)	Not reported	Not reported	Not reported	A VHA project in which a social worker trained in palliative care, taught stress reduction to carers through individual counselling and virtual groups	Low
22	Berwig (2020), Germany [51]	Evaluated the feasibility of Marte Meo® counselling with people with bvFTD and their primary carers	Mixed-methods; Qualitative component involved a telephone interview. Content analysis (Kuckartz, 2012), with inductive and deductive approaches	5 dyads (primary carer and PLWD): Spousal carers (*n *= 4), Adult child carers (*n *= 1)	Carers: All female PLWD: Female (*n *= 1), Male (*n *= 4)	Carers: 62.2 PLWD: 60	Not reported	All bvFTD	Not reported	Marte Meo® counselling is a video-based intervention for PLWD and carer dyads, that aims to maintain or improve the quality of dyadic relationships, and focuses on dialogue and interactions. The method uses video feedback as an indirect intervention, and the focus of MM counselling is the analysis and (co-)design of dialogues that involve communication and interaction processes	Low
23	Brännström (2000), Sweden [52]	Experiences of counselling groups for spouses of elderly PLWD	Qualitative; Semi-structured interviews. Grounded theory approach “followed closely” (Strauss and Corbin, 1990)	18 spousal carers	Female (*n *= 10), Male (*n *= 8)	Range of 55–76 yrs (mean not reported)	Not reported	Not reported	Not reported	Counselling groups that includes education about dementia and offers carers emotional and practical support for their daily living related to, for example, dementia-related behaviour, dementia-related problems of daily life such as household tasks, care giving and care arrangements	Low

*Abbreviations*: *PLWD* People living with dementia, *AD* Alzheimer’s disease, *VD* Vascular Dementia, *DwLB* Dementia with Lewy Bodies, *FTD* Frontotemporal dementia, *bvFTD* Behavioural variant frontotemporal dementia, *ACT* Acceptance and Commitment Therapy, *CBI* Cognitive Behavioural Intervention, *CBT* Cognitive Behavioural Therapy, *MIT* Mentalizing Imagery Therapy, *MBCT* Mindfulness-Based Cognitive Therapy, *MBSR* Mindfulness Based Stress Reduction, *M* Mean, *GAD-7* Generalised Anxiety Disorder-7 [53], *PHQ-9* Patient Health Questionnaire-9 [54], *FCNs* Faith Community Nurses, *VHA* Veterans Health Administration

### Quality assessment

Five studies were designated “high” quality, nine as “medium” quality, and nine as “low” quality. None fulfilled all of the criteria in the revised qualitative checklist [26, 28]. See Appendix C and D for details.

### Thematic synthesis

Findings were organised into five broad themes (Table 2), hierarchically ordered as therapy context, positive therapy experiences, types of change experienced, negative therapy experiences and impact of wider societal contexts and events on experiences. Direct participant quotes are italicised within quotation marks, whereas author interpretations are shown within quotation marks but without italicisation. Unique study IDs are included after quotes in order to link individual quotes to the relevant research study.

**Table 2:** Overview of themes

Theme	Subtheme
1. Therapy context	• Mental health difficulties • Relationship changes between the carer and PLWD
2. Positive therapy experiences	• Therapist factors and therapeutic relationship (listened to and understood; safe space; therapist as neutral and non-judgemental; therapist/instructor’s style and voice; relationship bridging) • Social support: Group setting, social linking and signposting to community resources (learning facilitated in group setting; feeling less alone; social linking and social support; signposting to community resources) • Specific strategies helpful (including breathing techniques; specific ACT-based skills; written material; communication and caregiving skills; goal setting)
3. Types of change experienced types of change	• Increased awareness of one’s present emotions and needs • Increased focus on self-care and self-compassion • More able to reach out for help from network and community resources • (Change in) Perspective: better understanding and increased acceptance of dementia and their caregiving situation • More able to cope with caregiving challenges • Changes in mood and emotion regulation
4. Negative therapy experiences, suggestions and further needs	• Specific unhelpful experiences of therapy and suggestions for improvement (initial distrust of strangers, too much self-disclosure; not personalised enough; access to content in-between sessions; more or longer sessions; better consideration of size of groups and confidentiality; lack of therapists with understanding/awareness of dementia) • Further needs (need for a range of support in particular practical support; continued access to psychological therapy or support group options; more information on dementia progression and how to cope; timing of therapy important especially early intervention)
5. Impact of wider societal contexts and events	• Impact of the COVID-19 pandemic: Emotional distress and elevated anxiety, fear, stress and frustration • Intersection of the pandemic, class and ethnicity: accessibility of support that requires technology, and subsequent increased isolation among ethnic minority participants • Difficulties with applicability of specific techniques during pandemic

## Therapy context

### Mental health difficulties

Studies commonly reported mental health difficulties that were expressed by carers. These included, but were not limited to: anxiety and stress; carer burden; feelings of “guilt”^8^; lack of support from their network; feeling “burnt-out”^2^, worn-out or “overwhelmed”^14^; depression; loneliness and isolation; lack of time for self and/or other commitments; frustration and temper; caregiving affecting carers’ physical health; and disturbed sleep. A few studies also described that carers were concerned about reaching out to their network because they did not want to "burden them further”^7^.

### Relationship changes between the carer and PLWD

Three studies reported changes in the relationship between the carer and the PLWD after their diagnosis of dementia (for both children and spousal carers), including. “a reversal of previous roles”^5^, a change “from daughter or son to an almost parental role”^7^, “a role change in the marriage”^3^, or a loss of shared identity.

## Overall positive or helpful experience of therapy

The majority of studies reported an overall positive or helpful experience of the intervention being examined. For example:

*“What a great program. I would have thrown in the towel had it not been for my counsellor…”*^15^

### Therapist factors and therapeutic relationship

All studies rated as “high” quality, and more than 10 others, described that participants attributed positive experiences or benefits of therapy to therapist/instructor factors and the therapeutic relationship. Specifically, carer participants felt listened to and understood in eight studies^1,5,7,9,10,16,19,21^ (e.g. “feelings of being listened to by someone and reassurance from their therapist that the way they were feeling was normal among carers.”^1^) and they felt that it was a *“confidential”*^5^ space and that the therapist was *“someone neutral”*^5^, “impartial”^7^ and non-judgemental.

Furthermore, the therapist/instructor’s style and voice or *“talk and speech”*^4^ was specifically mentioned in the studies that used a mindfulness approach [35, 42, 48], CBI [32, 33, 47] and other approaches [43, 45, 46, 50, 52].

Two studies (one “high” and one “medium” quality), both counselling approaches, highlighted the importance of the therapeutic relationship and described the therapeutic relationship as “bridging”^5^ following “changes”^5^ in their relationship with the PLWD, or “essential as a support system that may fill the void that some participants may feel, resulting from an absent support network”^7^. Also, two studies (one “medium” quality and one “low”) discussed having knowledge of dementia and its profession and impact as being helpful:

“The fact that the counsellors were competent nurses with experience and interest in nursing (PLWD) was judged by the participants to be a necessary part of the programme and crucial for its successful outcome.”^23^

### Social support: Group setting, social linking and signposting to community resources

Eight studies^4,8,11,12,13,17,19,23^ (one “high” and seven “medium” quality) that offered therapy, or a component of their intervention, in a group setting illustrated that participants appreciated hearing and learning from others and that the group setting facilitated learning and social support, and felt “camaraderie”^11^, “fellowship”^23^, “connected”^11^ and less “alone”^11,8^. Aside from studies where the intervention consisted of a group component, social linking and social support from the therapist or intervention itself was also discussed in three studies^1,3,5^ and might highlight the importance and usefulness of enabling this within therapy. This contributed to the normalisation of participant’s difficulties and left them feeling less alone.

*“Knowing that there were people all over the country experiencing this and dealing with this and listening to the same programme that I was listening to, it did make me feel part of a community. […]*^1^

Five studies^3,8,9,16,21^ (one “high” quality, two “medium” and two “low”) reported finding signposting to community resources was (or could potentially be) helpful:

*“I have become confident about the situation. I know what to do when the different situations occur, I know who to contact, and that is a great help, and if there is something I don’t know I can look it up in my book.”*^3^

“When caregivers needed outside assistance and had limited access to it, it was helpful for the FCNs (faith community nurses) to introduce the caregivers to available resources for home care help, such as church respite programs, adult day cares, support groups, and alternatives to ALFs (assisted living facilities). A caregiver described the outside help as *some light at the end of the tunnel.’*”^9^

### Specific strategies were helpful

Studies reported different specific strategies that participants found helpful, and the common ones are reported below.

Breathing techniques were described as helpful in five studies^4,10,11,18,20^, including three that used mindfulness approaches^4,11,18^; this contributed to carers feeling more present and relaxed in the moment:

*“It brings you back to the here and now. It puts things in perspective for me: It’s not as bad as you think it is. When I feel myself tense up, I do the breathing. That really helps me.”*^18^

Specific ACT-based skills were reported to be helpful from two studies reporting on the same iACT4 CARERS intervention [13, 31], and these techniques included present moment focus and noticing emotions; the carer case example (“Mrs Stewart”) was normalising and validating of their experiences and “seeing Mrs Stewart overcoming challenges using ACT skills was inspiring”^1^; and metaphors were helpful memory aids of previously learnt therapy skills..

Written material, relevant for three studies^11,19,21^, was reported by participants as “helpful”^21^ and could “lower our stress”^21^ (one medium and two low quality).

*“It's just been an awesome tool, it's just like turning on the light. You knew all this stuff was here, you already had this information. But it was just like you were walking around in the dark. But now I have the lights on, and I'm opening the book and pulling the tools out, instead of digging the ground with my hands, I am using the tools.”*^19^

A range of communication and caregiving skills were also reported to be helpful in three studies^10,17,20^ (one medium and two low quality) in order to improve communication between carer and PLWD which “led to reductions in frustration and disagreements”^17^. These changes varied between “stop and think”^17^ before speaking with/reacting to PLWD, being more assertive, increased interactions between the dyads, and “companionate communication”^10^ skills with PLWD.

Goal setting was also reported to be helpful for two interventions, both CBI and faith-based (three studies^13,17,19^, two of which reported on the same intervention), which supported participants to resume past social activities and hobbies.

## Types of change experienced

### Increased awareness of one’s present emotions and needs

Ten studies^1,3,5,7,9,11,13,17,19,20^ (three “high” quality, four “medium” and 3 “low”; and various approaches of therapy) mentioned an increased awareness of one’s present emotions and needs following taking part in therapy, and for some studies this included finding balance between their own needs and PLWD’s needs:

*“I might not have been as aware of my own needs if [FCN] had not pointed out the importance.”*^9^

*“So he’s helping me focus on me, and trying to help me, myself, rather than what I normally do, helping other people.”*^5^

The specific ways in which the therapy contributed to this varied across the studies. For example, illustrated from the three mindfulness-based studies^4,11,20^, being more present in the moment and deep breathing led to feeling more relaxed or calm; goal-setting enabled this for Glueckauf et al.’s [33] CBI study; for Griffiths et al. [38] awareness was facilitated through reflecting with the therapist; for Yang et al. [43] this was enabled through mentalising own’s present emotions; and for Contreras et al. [13] and Kazmer et al. [40] increased awareness was reported as a result of the intervention or therapist generally helping carers to understand the importance of self-care and one’s own needs.

### Increased focus on self-care and self-compassion

Building on from an increased awareness of one’s own needs and emotions, 11 studies^1,2,3,7,9,11,12,13,17,19,23^ (three “high” quality, 5 “medium” and 3 “low”) also reported an increased focus on self-care and self-compassion (increased understanding of importance of self-care) and the importance of taking care of oneself in order to care for others:

*“I know now that in order to care for someone else, you've got to take care of yourself”*^19^

This was closely linked to increased self-compassion in one study:

“Self-compassion was an area commonly mentioned by participants [...] *‘I’m much more compassionate toward myself…forgiving myself when I’m not at 100%.’*”^12^

Similar to the subtheme above, the way in which this was carried out varied across studies. For example, in Contreras et al. [13, 31] and Griffiths et al. [38], the importance of taking care of yourself in order to care for others appears to have been explicitly mentioned (e.g. *“as the time went on and she went through the programme and learnt actually it’s so important to care for herself, as well as caring for others”*^2^); in the CBI studies this involved goal-setting as well; and in Sørensen et al. [34] participants referred to feeling more able to take a break when needed (and more awareness of when a break is needed):

*“I have also learned how to take care of myself and how to preserve my energy. I have become more pragmatic, and I don’t feel guilty when I need a break, so I take it when I can feel I’m getting exhausted”*^3^

### More able to reach out for help from network and community resources

Seven studies^2,8,9,13,15,17,23^ (including one “high” quality) reported this subtheme. This was also linked to asking for help from their network “without feeling too guilty for doing so”^2^. Another study highlighted that counselling “aided them (carers) in asking for help”^15^. Glueckauf et al. [33, 47] also spoke about how “assertiveness training”^13^ facilitated reaching out “on others for assistance and to negotiate anxiety-provoking exchanges with family members reluctant to provide support”^13^. This also led to an increase in quality time with their network for Griffith et al. [38].

### (Change in) Perspective: Better understanding and increased acceptance of dementia and their caregiving situation

Seven studies^3,7,8,12,18,21,23^ (one “high”, five “medium” and one “low” quality) reported that the intervention facilitated an increased awareness and better understanding of dementia symptoms/impacts. For two studies [38, 39], this led to a change in perspective and an increased acceptance of dementia and their situation. For example:

*“I feel more accepting of things, of my situation. I feel more upbeat about it”*^12^

“Participants commonly spoke of increased self-acceptance and acceptance of family members’ illnesses following mindfulness training, coupled with being less judgmental of both self and family member. [...] *“There’s peace because there’s nothing that can be done. You quit trying to fix something that can’t be fixed. There is no peace without acceptance.”*”^18^

### More able to cope with caregiving challenges

Linked to the above, three studies^3,8,20^ (one “high”, one “medium” and one “low” quality) indicated that following the intervention, their participants reported feeling “better able to cope with the challenges”^3^ involved in dementia caregiving. This was attributed to having a better understanding of dementia symptoms, consequences and what to do when different situations occur, managing behavioural problems, and increased acceptance of dementia.

### Changes in mood and emotion regulation

Changes in mood, emotional state or mental health were reported by a number of studies, for example:

“Although caregivers felt frustrated, anxious, or depressed during the early COVID-19 pandemic and “Stay-at-Home” order period, they reported that their emotional distress decreased and became more positive after receiving telephone support.”^10^

A common experience reported by two studies^3,8^ was a reduction in guilt. Three studies^3,7,8^ (one “high” quality and two “medium”) also reported a change in perspective that contributed to positive changes in mood. Feeling more relaxed was also reported in a previous theme in relation to the contribution of breathing exercises. Berk et al. [42] reported that one participant felt sadness during the mindfulness training due to being more aware of present emotions when practicing the techniques, but that this was not perceived negatively.

## Negative therapy experiences, suggestions and further needs

### Specific unhelpful experiences of therapy and suggestions for improvement

Two studies^7,9^ (one “high” quality and one “medium”) referred to initial distrust of strangers, and that trust was built with their therapist over time:

“[…] establishing a connection was initially challenging for other pairs, as some caregivers were reluctant to receive help, did not feel comfortable letting a stranger into their lives, or did not understand exactly how the program could help them.[...] *‘The first couple of weeks it was like, this strange person is coming in, and [CG (carer)] is not sure what I’m doing, and I’m not sure what [CG] wants, but each week I feel more of a connection.’*”^9^

Elvish et al. [36] also reported that excessive self-disclosure from therapists was deemed unhelpful by participants and “crossed too many boundaries”^5^, and that the therapist’s feedback was “not personalised enough”^5^ and “did not resonate with them”^5^. Contreras et al. [13] also mentioned wanting access to content in-between sessions to remind themselves of what they asked. Tahsin et al. [35] outlined that participants would prefer more or longer sessions, as opposed to the one-off mindfulness session that they received.

Johanessen et al. [39] provided a range of suggestions for improvement: related to the intervention format, they highlighted a need for less structure and more open-discussion; and offering young-onset dementia carers “their own groups, because they were in another life situation and were facing other kinds of challenges”^8^, better consideration of size of groups and confidentiality, “in smaller municipalities where everyone knows each other, it is perhaps better to offer individual sessions”^8^.

Brooks et al. [37] also highlighted that there was a lack of therapists with an understanding or awareness of dementia, which can be seen as a barrier to attending therapy. More broadly, a need for therapists or healthcare staff with sufficient knowledge of dementia was suggested.

“Psychological support was sought by some spouses experiencing high levels of grief or depression; however, it was felt that most counsellors or psychologists did not have sufficient knowledge of dementia to provide optimal support: *‘I just found it really difficult to get that fit of someone who understood the nuances of dementia’*”^6^

### Further needs

In addition to suggestions for improvement for specific interventions, five papers^3,7,8,16,19^ (including one “high” quality, two “medium” and two “low”) illustrated further expressed needs of carers who have accessed therapy and expectations of quality of therapy. Specifically, this included a need for a range of support signposting (e.g. “need for comprehensive support provided by a range of people”^7^), in particular practical support:

“Help with applications (incl. health insurance); Counselling about support offers; Counselling in financial matters; Help (in general)”^16^

Three studies^6,9,16^ reported a need for more information on dementia progression including “how to anticipate and deal with care partners’ degeneration”^9^, and “information relating to long-term care provision.”^6^

(Continued) access to psychological therapy, “peer support and dementia-specific grief counselling”^6^ or “caregiver support groups”^19^ options were described as a need throughout the course of dementia in four studies^3,6,16,19^ (including one high quality).

Moreover, the timing of when therapy was offered was deemed important in two studies, in particular the importance of early intervention was highlighted; and that this offer should be initiated by professionals rather than carers/PLWD “having to seek it themselves”^6^. Brooks et al. [37] and Johanessen et al. [39] also recommended care/nursing home “transitional counselling”^6^ support is offered to carers, which could indicate a particularly stressful and vulnerable period for carers.

## Impact of wider societal contexts and events

Two more recent studies^1,10^ discussed the impact of the COVID-19 pandemic on carers’ mental health and therapy provision. Lee et al. [41] showed how the COVID pandemic caused emotional distress among carers (fear of the virus and infecting PLWD), and being stuck at home providing “around-the-clock care” during lockdown as well as the loss of outside support and respite activities (e.g. closing of day-care centres, limiting contact with family/friends) led to “forced social isolation” and"strong feelings of disconnectedness, loneliness, and frustration”.

Lee et al. [41] highlighted the intersection of the pandemic, class and ethnicity on the accessibility of support that requires technology, and subsequent increased isolation among ethnic minority participants:

“There were differences in technology access among diverse caregivers in our study. Most English-speaking participants expressed they were able to build social connection using technology (e.g., Zoom) with their family members and/or significant others, while many ethnic minority caregivers (i.e., Latinx, Korean, and Vietnamese) reported that they had limited technology and/or internet access. Thus, they felt even more isolated during this “Stay-at-Home” order period”^10^

Contreras et al.’s [31] participants discussed difficulties with the applicability of specific techniques in the intervention during the pandemic and restrictions at the time, and that the pandemic “hindered the opportunity of putting the learned skills into practice”^1^ and thus impacted on the usefulness of the intervention.

## Discussion

This study is the first to our knowledge to systematically review qualitative research on the experiences of carers of PLWD in accessing and attending psychological therapies. Findings from 23 studies suggested that the provision of psychological therapies for carers involves a range of therapy modalities. There were predominantly overall positive or helpful experiences of therapy reported, as well as positive changes in mood after completion of therapy. A number of mechanisms of change are outlined, including increased focus on self-care and self-compassion. Unhelpful therapy experiences, suggestions for improvement and further needs of this population were also reported.

One of the most commonly mentioned impact of therapy was an increased awareness of one’s present emotions and needs, as well as increased focus on self-care and self-compassion. Given previous research indicating that caregiving affects carers’ physical, mental and social self-care and they have less time to take care of their own health [55–58], it appears that interventions which target awareness of carers’ own needs and self-care, could be beneficial. This aligns with previous research on self-compassion, which found that carers with higher levels of self-compassion reported lower levels of caregiver burden, and this could partially be due to the use of less dysfunctional coping strategies [59].

The findings also highlighted the importance of social support and how social linking or signposting to community resources could be helpful for dementia carers. Third-wave CBT techniques, particularly breathing techniques and mindfulness, also appear to be especially beneficial.

The impact of the COVID-19 pandemic and how this might vary according to carers’ class and ethnicity outlines social inequalities within this context, including one specific example reported by Lee et al. [41] whereby being an ethnic minority could lead to increased isolation due to limited access to technology.

This review illustrates that taking part in certain types of psychological therapies can be positively experienced by, and beneficial for, carers of PLWD, and suggests that they may be acceptable to this population. This is in line with systematic reviews and meta-analyses indicating that psychosocial therapies, such as multicomponent interventions, CBT and mindfulness-based therapies, can have small-to-moderate effects on carer burden, depression and general health [10, 21, 60].

### Limitations

The strengths of this review are in the rigorous search and synthesis of literature according to recommended methodologies and tools. Limitations include the heterogeneity of therapy type (e.g., CBT, counselling) and modalities (e.g., group, individual) of included studies. This diversity might make it difficult to generalise findings across different interventions. However, a key finding was that, regardless of approach, therapist factors (e.g., non-judgemental, good listener) and group processes (e.g., normalisation, camaraderie) were important. The current study excluded interventions that consisted of support groups or psychoeducation only which means that these were not represented and may limit the generalisability of findings. Furthermore, for multi-component interventions that were included, it is difficult to know exactly what aspect was helpful, and for whom.

From the included studies, only one (quality rated as low) reported time constraints of dementia care as a barrier to attending a component of their intervention [45]. No other studies mentioned difficulties finding time as a barrier to attending therapy, and no studies reported concerns about leaving the PLWD as a barrier to engagement with services. One study reported busyness and unpredictability of carers’ schedules as a barrier to scheduling an interview for their qualitative study, but not as a barrier to engaging in the therapy/intervention itself [32]. This could indicate a sampling bias within the included studies, as perhaps carers who could not find time to engage in the psychological interventions might not have taken part in the study or might have dropped out.

Studies also varied in quality, with only five of 23 rated high. Thus, methodological rigour was variable, including factors such as sample selection, lack of reflexivity, and/or lack of transparency of data analysis methodology, which could undermine the credibility of the synthesised results. Moreover, as only 10% of the titles/abstracts and full texts were dual screened, this might have introduced bias in terms of inclusion and exclusion of studies. A further limitation was the exclusion of non-English language studies, which means that potentially relevant studies in other languages might have been excluded. This could result in a language bias, as well as potentially incomplete evidence synthesis and conclusions.

### Direction for future research

The low number of “high” quality research studies in this context highlights the importance of conducting higher-quality qualitative research on the topic to enhance the rigor and credibility of future syntheses in this area, as well as to fill the gaps in knowledge and strengthen the evidence base. For example, ensuring that the research design and methodology is justified; that the data analysis process is described transparently and in sufficient detail and that the researcher has adequately and critically considered their own role and potential bias during the research process; and that there is sufficient information that demonstrates clear, consistent and conceptually coherent research design, analysis methods and theoretical underpinnings [26, 28].

Future research could include detailed exploration of the specific elements of different psychological therapies which are most effective in this population, as well as for different dementia types and at different points of dementia progression. This should be done with the aim of adapting existing therapies and/or creating new ones which incorporate techniques across the different modalities outlined above, such as emphasising social and/or group aspects of therapy or including third-wave techniques. Importantly however, therapies should focus on person-centred care and meeting individuals’ specific needs, rather than treating them as a uniform group and carers should be involved in such research through co-production. Another need is to understand for whom and under what circumstances interventions become most effective (e.g. spousal carers versus adult child carers; when during disease progression; which type of dementia) and to examine contextual and implementation mechanisms underlying psychosocial interventions. Tailoring interventions to the specific needs of caregivers is crucial to making them most effective.

### Implications

This review suggests that therapy overall is helpful and makes recommendations to: 1) tailor therapies regardless of their approach, 2) point to outcome measures we should be focusing on (e.g. those that measure self-compassion or social support), and 3) identify techniques (e.g. increasing self-compassion) that are helpful regardless of approach. In identifying therapy outcomes that are important to carers, the findings are useful to think about what to focus on in future effectiveness studies. In identifying therapist factors and unhelpful aspects of therapy, findings might be helpful in tailoring therapy (regardless of approach) to best meet carers’ needs. In thinking about specific techniques that are helpful, the results supplement quantitative data about what might be useful to focus on in future interventions. In considering intersectionality of class, ethnicity and global events, the findings start to unpick where psychological therapies might not be serving people and appropriately considering their wider context, which the MRC framework [22] highlights as being important to do, as well as where there may be unmet or a mismatch of needs and access issues for carers.

A key subtheme highlighted was the need to address caregivers’ physical and psychological health and needs, and the importance of self-care and self-compassion, within both physical and mental health-care services. This indicates that potentially where able, healthcare services need to take an active approach to ensuring carers attend their routine appointments or regular health check-ups.

In conjunction with the conclusions of systematic reviews and meta-analyses which demonstrate tentative evidence for the use of psychological therapies with carers of PLWD [21, 60], the current results suggest that services, clinicians and policy should be informed by the potential benefits of these interventions for this population. To attain such benefits, increased funding and service flexibility might be crucial. Moreover, offering greater support for clinicians to participate in dementia awareness training, along with the time and resources to adapt existing therapies, could enhance accessibility and lead to wider positive outcomes for carers of PLWD. It suggests that tailoring therapies to the specific needs of caregivers is imperative to making them most effective, for example perhaps that there might be different therapy needs for carers of different types or stages of dementia. Digital interventions might potentially exclude a group of individuals and so if offering this, such accessibility considerations should first be taken into account.

## Conclusions

The current review demonstrates predominantly overall positive experiences and impacts of therapy, indicating that therapy can be helpful for this population. It suggests specific techniques that can help tailor therapy (regardless of approach) to best meet carers’ needs and highlights the importance of considering wider contextual factors and aspects of identity that can influence carers’ experience of and access to therapy. There were also specific unhelpful experiences of therapy and suggestions for improvement reported. Future research should include higher-quality qualitative research on this topic. It is important to understand for whom and under what circumstances interventions become most effective and examine contextual and implementation mechanisms underlying psychosocial interventions.

### Clinical trial number

Not applicable.

## Supplementary Information

Supplementary Material 1

## Data Availability

No datasets were generated or analysed during the current study.
