# Supplementary material for: A systematic review and thematic synthesis on the experiences of accessing and attending psychological therapy for informal carers of people living with dementia
Source: BMC Geriatr. 2025 May 19;25:353. doi: 10.1186/s12877-025-05986-7 (PMC12087096; doi:10.1186/s12877-025-05986-7)
Supplement: Supplementary file 1 — Supplementary Material 1 [file 12877_2025_5986_MOESM1_ESM.docx]

**Supplements**

**Appendix A: Systematic Review Search Strategy**

**Web of Science**

TI=(dementia* OR Alzheimer* OR Huntington* OR Lewy Bod* OR Parkinson*) OR AB=(dementia* OR Alzheimer* OR Huntington* OR Lewy Bod* OR Parkinson*)

*AND*

TI=(caregiver* OR care‐giver* OR "care giver*" OR carer* OR dependents OR families* or family* or folk* or kinship or parent* OR relatives OR spouse* OR wife* OR wives* OR husband*) OR AB=(caregiver* OR care‐giver* OR "care giver*" OR carer* OR dependents OR families* or family* or folk* or kinship or parent* OR relatives OR spouse* OR wife* OR wives* OR husband*)

*AND*

TI=(psychotherap*) OR AB=(psychotherap*) OR TI=(“acceptance and commitment therapy”) OR AB=(“acceptance and commitment therapy”) OR TI=("cognitive behavioural therapy" OR “cognitive behavioral therapy” OR “cognitive behaviour therapy” OR “cognitive behavior therapy” OR “CBT” OR “cognitive therapy”) OR AB=("cognitive behavioural therapy" OR “cognitive behavioral therapy” OR “cognitive behaviour therapy” OR “cognitive behavior therapy” OR “CBT” or “cognitive therapy”) OR TI=("talking therapy" OR “talking therapies”) OR AB=("talking therapy" OR “talking therapies”) OR TI=(mindful*) OR AB=(mindful*) OR TI=(“compassion focused therapy”) OR AB=(“compassion focused therapy”) OR TI=(counselling OR counseling) OR AB=(counselling OR counseling) OR TI=(IAPT OR “improving access to psychological therapy” OR “improving access to psychological therapies”) OR AB=(IAPT OR “improving access to psychological therapy” OR “improving access to psychological therapies”) OR TI=(psychological therap*) OR AB=(psychological therap*) OR TI=(guided self help) OR AB=(guided self help) OR TI=("couple therapy" or "couples therapy") OR AB=("couple therapy" or "couples therapy") OR TI=(psychodynamic) OR AB=(psychodynamic) OR TI=(psychoanalysis) OR AB=(psychoanalysis) OR TI=(EMDR OR “eye movement desensitisation and reprocessing”) OR AB=(EMDR OR “eye movement desensitisation and reprocessing”) OR TI=(DBT OR “dialectical behaviour therapy” OR “dialectical behavior therapy”) OR AB=(DBT OR “dialectical behaviour therapy” OR “dialectical behavior therapy”) OR TI=(REBT OR “rational emotive behaviour therapy”) OR AB=(REBT OR “rational emotive behaviour therapy”) OR TI=(“interpersonal therapy”) OR AB=(“interpersonal therapy”) OR TI=("compassion focused therapy" OR "compassion-focused therapy") OR AB=("compassion focused therapy" OR "compassion-focused therapy") OR TI=(“behavioural activation” OR “behavioral activation”) OR AB=(“behavioural activation” OR “behavioral activation”)

**CINAHL**

( TI ( dementia* OR Alzheimer* OR Huntington* OR Lewy Bod* OR Parkinson* ) OR AB ( dementia* OR Alzheimer* OR Huntington* OR Lewy Bod* OR Parkinson* ) ) AND ( TI (carer* OR caregiver* OR care‐giver OR spouse‐caregiver* OR “care giver” OR dependents OR families* OR family* OR folk* OR kinship* OR parent* OR relatives OR spouse* OR wife* OR wives* OR husband*) OR AB (carer* OR caregiver* OR care‐giver OR spouse‐caregiver* OR “care giver” OR dependents OR families* OR family* OR folk* OR kinship* OR parent* OR relatives OR spouse* OR wife* OR wives* OR husband*) ) AND ( TI ( psychotherap* OR “psychological therap*” OR “guided self help” OR “behavioural activation” OR “behavioral activation” OR “couple therapy” OR “couples therapy” OR psychodynamic OR psychoanalysis OR EMDR OR “eye movement desensitisation and reprocessing” OR DBT OR “dialectical behaviour therapy” OR “dialectical behavior therapy” OR REBT OR “rational emotive behaviour therapy” OR “rational emotive behavior therapy” OR “interpersonal therapy” OR "compassion focused therapy" OR "compassion-focused therapy" OR “acceptance and commitment therapy” OR "cognitive behavioural therapy” OR “cognitive behavioral therapy” OR “cognitive behaviour therapy” OR “cognitive behavior therapy” OR CBT OR "talking therapy" OR “talking therapies” OR mindful* OR “compassion focused therapy” OR counselling OR counseling OR IAPT OR “improving access to psychological therapy” OR “improving access to psychological therapies” ) OR AB ( psychotherap* OR “psychological therapy” OR “guided self help” OR “behavioural activation” OR “behavioral activation” OR “couple therapy” OR “couples therapy” OR psychodynamic OR psychoanalysis OR EMDR OR “eye movement desensitisation and reprocessing” OR DBT OR “dialectical behaviour therapy” OR “dialectical behavior therapy” OR REBT OR “rational emotive behaviour therapy” OR “rational emotive behavior therapy” OR “interpersonal therapy” OR "compassion focused therapy" OR "compassion-focused therapy" OR “acceptance and commitment therapy” OR "cognitive behavioural therapy” OR “cognitive behavioral therapy” OR “cognitive behaviour therapy” OR “cognitive behavior therapy” OR CBT OR "talking therapy" OR “talking therapies” OR mindful* OR “compassion focused therapy” OR counselling OR counseling OR IAPT OR “improving access to psychological therapy” OR “improving access to psychological therapies” ) )

**PsycINFO**

1 exp dementia/

2 (dementia* or Alzheimer* or Huntington* or Lewy Bod* or Parkinson*).ti,ab.

3 exp Caregivers/ or exp Family/ or exp Family Members/ or (caregivers or care giver* or carer* or caregiv* or care-giver* or spouse-caregiver* or dependents or families* or family* or folk* or kinship or parent* or relatives or spouse* or wife* or wives* or husband*).ti,ab.

4 (mental health adj2 (therap* or psychotherap*)).ti,ab.

5 (mental illness adj2 (therap* or psychotherap*)).ti,ab.

6 (psychopathology adj2 (therap* or psychotherap*)).ti,ab.

7 (psychiatric adj2 (therap* or psychotherap*)).ti,ab.

8 (psycholog* adj2 therap*).ti,ab.

9 mindful*.ti,ab.

10 (IAPT or improving access to psychological therapy or improving access to psychological therapies).ti,ab.

11 "acceptance and commitment therapy".ti,ab.

12 psychotherapeutic counseling/

13 counseling/ or community counseling/ or counselling.ti,ab.

14 exp Cognitive Behavior Therapy/ or ("cognitive behavio?r therapy" or "CBT").ti,ab.

15 "guided self help".ti,ab.

16 "behavio?ral activation".ti,ab.

17 ("couple therapy" or "couples therapy").ti,ab.

18 psychodynamic.ti,ab.

19 psychoanalysis.ti,ab.

20 (EMDR or "eye movement desensitisation and reprocessing").ti,ab.

21 (DBT or "dialectical behavio?r therapy").ti,ab.

22 (REBT or "rational emotive behavio?r therapy").ti,ab.

23 "interpersonal therapy".ti,ab.

24 ("compassion focused therapy" or "compassion-focused therapy").ti,ab.

25 ("talking therapy" or "talking therapies").ti,ab.

26 1 or 2

27 4 or 5 or 6 or 7 or 8 or 9 or 10 or 11 or 12 or 13 or 14 or 15 or 16 or 17 or 18 or 19 or 20 or 21 or 22 or 23 or 24 or 25

28 3 and 26 and 27

**Appendix B: Quality Assessment Criteria Items used (Critical Appraisal Skills Programme, 2018; Long, French, & Brooks, 2020)**

1. Was there a clear statement of the aims of the research?
2. Is a qualitative methodology appropriate?
3. Was the research design appropriate to address the aims of the research?
4. Additional item (Long, French & Brooks, 2020): Are the study’s theoretical underpinnings (e.g. ontological and epistemological assumptions; guiding theoretical framework(s)) clear, consistent and conceptually coherent?
5. Was the recruitment strategy appropriate to the aims of the research?
6. Was the data collected in a way that addressed the research issue?
7. Has the relationship between researcher and participants been adequately considered?
8. Have ethical issues been taken into consideration?
9. Was the data analysis sufficiently rigorous?
10. Is there a clear statement of findings?
11. How valuable is the research? (open-ended question)

**Appendix C: Quality Assessment Ratings (Critical Appraisal Skills Programme, 2018; Long, French, & Brooks, 2020)**

| **Study ID** | **Authors** | **Year** | **Q2 - Is a qualitative methodology appropriate?** | **Q3 - Was the research**  **design appropriate to**  **address the aims of the**  **research?** | **Q9 - Was the data analysis sufficiently rigorous?** | **Overall quality score based on tipping point items** | **Rating descriptor** |
| --- | --- | --- | --- | --- | --- | --- | --- |
| 1 | Contreras, Van Hout, Farquhar, McCracken, Gould, Hornberger, Richmond, & Kishita | 2022 | Yes | Yes | Yes | 3 Yes | High |
| 2 | Contreras, Van Hout, Farquhar, McCracken, Gould, Hornberger, Richmond, & Kishita | 2021 | Yes | Yes | Yes | 3 Yes | High |
| 3 | Sorensen, Waldorff, & Waldemar | 2008 | Yes | Yes | Yes | 3 Yes | High |
| 4 | Tahsin, Stanyon, Sun, & Gamble | 2021 | Yes | Yes | Yes | 3 Yes | High |
| 5 | Elvish, Cawley, & Keady, | 2014 | Yes | Yes | Yes | 3 Yes | High |
| 6 | Brooks, Beattie, Fielding, Wyles, & Edwards | 2022 | Yes | Yes | Somewhat | 2 Yes & 1 Somewhat | Medium |
| 7 | Griffiths, Shoesmith, Sass, Nicholson, & Charura | 2020 | Yes | Yes | Somewhat | 2 Yes & 1 Somewhat | Medium |
| 8 | Johannessen, Bruvik, & Hauge | 2015 | Yes | Yes | Somewhat | 2 Yes & 1 Somewhat | Medium |
| 9 | Kazmer, Glueckauf, Schettini, Ma, & Silva | 2018 | Yes | Yes | Somewhat | 2 Yes & 1 Somewhat | Medium |
| 10 | Lee, Kim, Ju, Guo, Rousseau, Gibbs, Tran, Tom, Sabino-Laughlin, & Kehoe | 2023 | Yes | Yes | No | 2 Yes & 1 No | Medium |
| 11 | Berk, Warmenhoven, Stiekema, van Oorsouw, van Os, de Vugt, & van Boxtel | 2019 | Yes | Somewhat | Somewhat | 1 Yes & 2 Somewhat | Medium |
| 12 | Yang, Zamaria, Morgan, Lin, Leuchter, Abrams, Chang, Mischoulon, Pedrelli, Fisher, Nyer, Yeung, & Jain | 2023 | Yes | Somewhat | Somewhat | 1 Yes & 2 Somewhat | Medium |
| 13 | Glueckauf, Davis, Willis, Sharma, Gustafson, Hayes, Stutzman, Proctor, Kazmer, Murray, Shipman, McIntyre, Wesley, Schettini, Xu, Parfitt, Graff-Radford, Baxter, Burnett, Noël, Haire, & Springer | 2012 | Yes | Somewhat | Somewhat | 1 Yes & 2 Somewhat | Medium |
| 14 | Vernooij-Dassen, Joling, van Hout, & Mittelman | 2010 | Yes | Somewhat | Somewhat | 1 Yes & 2 Somewhat | Medium |
| 15 | Gaugler, Reese, & Mittelman | 2018 | Somewhat | Somewhat | Somewhat | 3 Somewhat | Low |
| 16 | Gräßel, Luttenberger, Trilling, & Donath | 2010 | Somewhat | Somewhat | Somewhat | 3 Somewhat | Low |
| 17 | Glueckauf, Kazmer, Nowakowski, Wang,  Thelusma, Williams, McGill-Scarlett, Lampe, Norton-Brown,  Davis, Sharma, & Willis | 2022 | Somewhat | Somewhat | Somewhat | 3 Somewhat | Low |
| 18 | Hoppes, Bryce, Hellman, & Finlay | 2012 | Can’t Tell | No | Somewhat | 1 Can’t Tell, 1 No, 1 Somewhat | Low |
| 19 | Kazmer, Glueckauf, Ma, & Burnett | 2013 | Somewhat | Somewhat | No | 1 No, 2 Somewhat | Low |
| 20 | Kor, Liu, & Chien | 2019 | Can’t Tell | Somewhat | Can’t Tell | 2 Can’t Tell, 1 Somewhat | Low |
| 21 | Koufacos, Gottesman, Dorisca, & Howe | 2023 | Somewhat | Somewhat | Can’t Tell | 2 Somewhat, 1 Can’t Tell | Low |
| 22 | Berwig, Dinand, Becker, & Halek | 2020 | Somewhat | Yes | No | 1 No, 1 Yes, 1 Somewhat | Low |
| 23 | Brännström, Tibblin, & Löwenborg | 2000 | Somewhat | Somewhat | No | 2 Somewhat, 1 No | Low |

**Appendix D: Quality Assessment Summary**

For most of the studies (N=18), their qualitative data analysis was not sufficiently rigorous enough to be considered a ‘yes’, and their analysis method and theoretical underpinnings were not deemed to be clear, consistent and conceptually coherent due to a lack of information provided. In line with this, the majority of studies did not (N=13) or only somewhat (N=7) adequately consider the relationship between researcher and participants, and for another study it was not clear whether they did so (‘can’t tell’). Furthermore, for the majority of studies (N=12) there was insufficient information to determine whether the research designs used were appropriate. Similarly, for six studies it was deemed that the detail provided was insufficient to assess whether the relationship between researcher and participants been adequately considered
